# HTLV-1 Tax Specific CD8+ T cells express low levels of Tim-3 in HTLV-1 infection: implications for progression to neurological complications

**DOI:** 10.1186/1742-4690-8-S1-A94

**Published:** 2011-06-06

**Authors:** Fabio E Leal, Lishomwa C Ndhlovu, Aaron M Hasenkrug, Aashish R Jha, Karina I Carvalho, Ijeoma G Eccles-James, Fernanda R Bruno, Raphaella G Vieira, Vanessa A York, R Brad Jones, Yuetsu Tanaka, Walter K Neto, Sabri S Sanabani, Mario A Ostrowski, Aluisio C Segurado, Douglas F Nixon, Esper G Kallas

**Affiliations:** 1 Division of Clinical Immunology and Allergy School of Medicine, Universidade de Sao Paulo, Brazil; 2 Department of Infectious Diseases School of Medicine Universidade de Sao Paulo, Sao Paulo, Brazil; 3 Division of Experimental Medicine, Department of Medicine, University of California, San Francisco, CA, 94110 , USA; 4 Department of Immunology , University of Toronto, Medical Sciences Building, King s College Circle, Toronto, ON M5S 1A8, Canada; 5 Department of Immunology, University of the Ryukyus, Okinawa, 903-0215, Japan; 6 Molecular Biology Laboratory, Fundação Pro-Sangue, Hemocentro de Sao Paulo, Sao Paulo, Brazil

## Background

Most patients with HTLV-1 infection are asymptomatic, however 3% of individuals develop a progressive neurological disorder, HTLV-1 associated myelopathy/tropical spastic paraparesis (HAM/TSP). Factors leading to this complication are not well defined, but are associated with high levels of pro-virus and cytolytic T cells. During chronic viral infections, virus-specific CD8+ T cells undergo altered patterns of differentiation and can become restrained from effector activity. We hypothesized that suppression of immune receptors T cell immunoglobulin and mucin domain-containing protein 3 (Tim-3) and PD-1 would result in partial reversal of a T cell restraint profile. In turn this increased cytolytic activity may mediate neuro-immunopathology of HAM/TSP.

## Methods

We investigated the expression of Tim-3 and PD-1 on T cells in 22 serially recruited HTLV-1 asymptomatic and HAM/TSP patients and 7 HTLV-1-seronegative matched controls, their distribution on HTLV-1-specific T cells and the relationship with T cell function.

## Results

Using flow cytometry, we found that patients with HAM/TSP had significantly lower levels of Tim-3+ PD-1- expressing CD8+ (p=0.002) and CD4+ (p=0.004) T cells compared to healthy uninfected controls. HTLV-1 Tax11-19, HLA-A*02 restricted CD8+ T cells among HAM/TSP individuals expressed markedly lower levels of Tim-3. Furthermore, we found that the frequency of Tim-3 expressing Tax11-19 specific CD8+ T cells inversely correlated with the number of IFN-γ secreting cells in response to the Tax Tax11-19 peptide.

## Conclusions

We propose that this low expression of Tim-3 on HTLV-1 Tax-specific T cells may lead to persistent and deleterious effector T cell pool, resulting in more inflammation and disease progression.

